# Is the philosophical construct of “habitus operativus bonus” compatible with the modern neuroscience concept of human flourishing through neuroplasticity? A consideration of prudence as a multidimensional regulator of virtue

**DOI:** 10.3389/fnhum.2014.00731

**Published:** 2014-09-18

**Authors:** Denis Larrivee, Adriana Gini

**Affiliations:** ^1^Educational Outreach Office, Catholic Diocese of CharlestonCharleston, SC, USA; ^2^Neuroradiology Division, Neuroscience Department, San Camillo-Forlanini Medical CenterRome, Italy

**Keywords:** neuroplasticity, synapses, virtues, prudence, Aquinas

## The classical and the contemporary: neuroplasticity and the reemergence of virtue

Unlike ancient Greece where personal virtue was the route to fulfillment, modern man typically seeks to improve human well-being by external means, in a process known as the medicalization of society. The apparent novelty of recent proposals in psychological theory to develop character strength, therefore, lies in their reemphasis on a personal implementation of positive values (Peterson and Seligman, 2004). Among the factors contributing to a new look at self-determination has been the capacity for the neural substrate to selectively alter itself via neuroplasticity. Indeed, the confluence of past and contemporary thinking may presage a consideration of neurobiological instantiation within which virtuous behavior may be enhanced in accord with principles governing neuroplastic change.

But what are virtues and positive traits? And to what extent can these conceptions inform our growing understanding of the neural contribution to human behavior? Presupposed in such questions is a conceptual ground needed to define a corresponding empirical terrain (Bennett and Hacker, 2003), without which such information would lack coherence and conclusive power. Accordingly, positive psychology identifies a positive trait as a “disposition to act, desire, and feel” involving the exercise of judgment and leading to a recognizable human excellence' (Park et al., 2004). The concise, but more precise formula of Aquinas, “habitus operativus bonus,” is similarly conceived (Hibbs, 1999). Anglicized, habitus connotes habit, often considered a compelling behavioral pattern reinforced through repetitive activity, but in an Aquinas context also evinces a freedom associated with the deployment of a skill acquired and honed through repeated engagement. Operativus, understood to mean operationally effective, connotes stability and continuity, a disposition to future performance. The third term, bonus, grants an orientational norm more precise than the analogous “recognizable excellence” and that Aquinas grounds in right reason and love of neighbor. Accordingly, we will employ the construct “habitus operativus bonus” rather than “positive traits” in the ensuing discussion.

A priori, habitus tacitly acknowledges a behavior's dependence on repetitive engagement. This acknowledgement has received much confirmation from empirical studies of patterned behavior; and many of the physiological, cellular, and molecular features have now been elucidated. Originally theorized by Hebb (1949) as an activity dependent synaptic strengthening, this interpretation was subsequently confirmed by Lomo's discovery (Lomo, 2003) of the long term potentiation effect (LTP). In the Hebbian scheme synaptic strength is enhanced by coincident, and repetitive, neural activity. The molecular details of this effect entail a host of short term, cell signaling and, when sufficiently stimulated, long term, transcriptional and cell restructuring mechanisms (Benfenati, 2007). The former involve an enhancement of Ca influx at both pre and post synaptic sites, together with a corresponding activation of Ca dependent protein kinases, lasting minutes to hours. The latter involve a wholesale restructuring of synaptic contacts that can potentiate enhanced synaptic efficiency for days and even months. A key mechanism in transcriptional up-regulation is the kinase mediated activation of the CREB set of activator and repressor proteins. The stabilization, and proliferation, of coordinated synaptic activity, thereby, increasingly routs information flow through select circuit pathways.

These observations confirm three conclusions that follow from the classic formulation. First, they show that habitual activity is needed to enhance synaptic strength. Second, the behavioral performance or skill is made more easily operative. The freedom spoken of by Aquinas is thus neurally provided for in the enhanced information flow through the behavioral circuit. Finally, the ease of flow facilitates and so disposes, the circuit to similar operation in the future.

Yet, what underlies the selection of one circuit in preference of another? In the classical formulation stress is laid on the learned features of virtuous behavior. In contemporary neuroscience patterned behaviors presuppose a unique circuitry also selected through learning. Insight into the underlying mechanisms of learning has come from studies of circuits comprised of small numbers of neurons. Two of these, habituation and sensitization, are particularly well understood. Habituation, described as the progressive decrease in amplitude or frequency of an output in response to external stimuli, restricts information flow from irrelevant stimuli by reducing Ca influx needed for presynaptic vesicle release (Rankin et al., 2009). Sensitization reverses this effect, and thereby emphasizes the impact of relevant stimuli, through a cAMP kinase induced increase in Ca. Nor are these alone. Recent studies of underlying mechanisms of associative learning reveal that molecular switches, such as insulin isoforms, for example, can alter the behavioral pattern in a stimuli dependent manner (Ohno et al., 2014). Such mechanisms, observed in simple systems, are likely to constitute unitary learning modules broadly used for more complex learning programs. The repertoire of cellular mechanisms, in fact, highlights the rich potential for the choreography of patterned routines in large scale networks (Neville et al., 2010). Learned behavior for what are undoubtedly large scale networks have now been demonstrated for motor skill acquisition (Dayan and Cohen, 2012), clinical therapy (Cramer et al., 2011), stress related responsivity (Davidson and McEwen, 2013), and language learning (Hosoda et al., 2013). Indeed, all behaviors for which there is a demonstrable need for repeated or habitual performance are likely to be undergirded by such plastic mechanisms, including the execution of virtue.

## Prudence and neuroscience in the pursuit of excellence

The manner of the selection of one circuit over another, nonetheless, has raised the question of the valuation of a behavior that may lead to its selection. It is in this context that the orientational construct of Aquinas, bonus, is pertinent. In what manner, then, does the granting of normative weight to virtue bear on neural function? Complicating the issue of value is the matter of valence, defined as compelling loci within the focal space that provoke sustained interest for attainment. Humans, as do all species, possess appetitive desires distributed over a broad range of physiological and cognitive demands (Berridge and Kringelbach, 2008). Given that their salience can vary over a particularly broad range, it is clear that individual variation can become extraordinarily diverse. To accommodate a full spectrum of valences, therefore they must be ordered hierarchically. How so? This is done in two ways. First, neuroplastic mechanisms may be understood as an innate capacity to prioritize values dispositionally through circuit reiterations. Secondly, they may be invoked experientially or intentionally through higher order processes. Experience dependent learning of motor skills in the cerebellum, and conditioning dependent learning in the hippocampus, for example, both implement other cortical centers, that progressively shift in a learning dependent manner (Melia et al., 1996; Doyon et al., 2002).

The very diversity of valences, however, and the relative intensities to which salience is attributed, generate transformations as numerous in kind as the individuals in whom they are effected. Accordingly, it is to other dimensions that recourse must be made to order what will ultimately become dispositional preferences. In a Thomistic scheme value is established rationally, according to the dictates of practical reason, i.e., prudentia, and by conformity to an ordering principle, i.e.,“beatitude.” The inclination to future performance, though, and the assessment of deviation from preferred behavior may be matters heavily influenced by neural architecture. There is, for example, a notable correspondence between habitual behavior, the computational properties of corrective learning algorithms, and the physiology/anatomy of the dopaminergic system, a correspondence that also appears to extend to goal directed behavior (Daw and Shohamy, 2008). Nonetheless, some values may be innate (Bloom, 2010), and others acquired (Stanley D Phelps and Banji, 2008) with little or no conscious reflection. Babies, who lack a power for speech, but who can still indicate intentions through eye movement, are able to discriminate between various actions as to their moral worth. They know, for example, when an action is unjust, or altruistic. This has been interpreted to indicate a native capacity for goodness in its “infancy.” Preconscious, implicitly acquired value, such as those studied with the implicit association test (IAT, 2014), likewise show that not all value is determined rationally.

Still, Aquinas' insistence on rational deliberation as the necessary, conscious precursor to normative assignments intrinsic to virtuous behavior, is receiving renewed neuroscientific interest. In value assignment studies of children, normative values were not conditioned by background attitudes, but rather by a structured rationale from which moral inferences were then drawn (Hussar and Harris, 2010). Moreover, the reflective process of deliberation is a manifestly and universal social tool (Bloom, 2010). Embedded in the recognition of such deliberation is the notion of its procedural development, according to logical inference and structured on grounding principles. Nevertheless, the practical reasoning spoken of by Aquinas, and by which prudence must be exercised, is very much in its infancy from the vantage of a neuroscientific understanding. Language, mental representations, syllogistic reasoning, insight, and relative judgments (Dadosky, 2014; Hauser et al., 2014) have stimulated theoretical discussion, but the empirical dimensions for which these concepts may antecede are at best correlative.

Perhaps most elusive is the manner in which the neural structure may be contributory to a state phenomenologically described by the orientational construct, bonus, the ordering principle by which normative assignments are inferred through rational and deliberative discourse. For Aquinas this first principle is self evident and non-deducible. Like habitus, this concept is also multitoned, expressing both the means that may be used, i.e., morality, as well as the goal, flourishing, or beatitude, that is to be attained. Such conceptions have been differentially interpreted neuroscientifically with most focus given to the practical means. A large body of work has now been devoted to how a moral understanding may have originated, usually couched in terms of its evolutionary, social development (Hauser, 2014). Rudimentary notions of altruistic behavior have been observed in some species (Zwick and Fletcher, 2014) and mirror neurons, which have been inferred to grant a capacity for empathic associations, studied (Rizzolatti and Craighero, 2004). Still the notion of beatitudo of Aquinas with its connotations of meaning, fulfillment, and openness to infinite and transcendental being does not appear capable of resolution at anything less than an integrationist account of the whole neural platform. Some suggestion of this appears in discussions of the neural underpinnings of the self and of downwardly causative operations for which the entire platform is likely to be necessitated or mobilized for (Sanguineti, 2013), but for the most part is undefined. Its relevance for a large part of humanity, though, is undeniable, propelling ongoing investigation. Prudentia thus has many aspects, and enters into every other virtue (Aquinas, 2011), the “form” shaping each virtue.

So how are we to view the utility of “habitus operativus bonus,” and the regulatory virtue of prudence in terms of conceptual schema for neuroscience? Born in a prebiological era Aquinas knew little of the functional elements of brain operation; yet he possessed an extraordinary analytical mind and a first person access to its events. Following a tradition traced to Aristotelian roots, Aquinas placed reason and will as the progenitors of behavior. Not so passion, Aquinas designation of emotion, despite his extensive and discrete analysis of the human emotional spectrum (Butera, 2010). Aquinas' observations, therefore, limited necessarily to those of whole systems, cannot be a guide to particular empirical events that underlie systems operations. Nevertheless, he recognized that such events are contributory, and in some cases determinative of their operation. It would have been no surprise that the virtue for which so much broad scale rational determination must be exercised would require so many subordinate neural circuits for its operation. And it would have been no surprise that to perfect its operation such circuits must be repeatedly deployed during learning, and in the process “disposed” and materially structured.

In his unified view of nature Aquinas offers landmarks circumscribing lower level events, the broad outlines of which serve in clarifying what the details must conform to, but not in identifying the materials by which the paths to these landmarks are structured. His integrationist accounts and objective philosophy of the purpose of brain operation, though, lay down a route of exploration likely to be increasingly relevant as neuroscience explores the global neural platform.

### Conflict of interest statement

The authors declare that the research was conducted in the absence of any commercial or financial relationships that could be construed as a potential conflict of interest.
